# Exercise leads to sex-specific recovery of behavior and pathological AD markers following adolescent ethanol exposure in the TgF344-AD model

**DOI:** 10.3389/fnbeh.2024.1448691

**Published:** 2024-08-01

**Authors:** Nicole L. Reitz, Polliana T. Nunes, Lisa M. Savage

**Affiliations:** Department of Psychology, Binghamton University, State University of New York, Binghamton, NY, United States

**Keywords:** alcohol, adolescence, exercise, Alzheimer’s disease, hippocampus

## Abstract

**Introduction:**

Human epidemiological studies suggest that heavy alcohol consumption may lead to earlier onset of Alzheimer’s Disease (AD), especially in individuals with a genetic predisposition for AD. Alcohol-related brain damage (ARBD) during a critical developmental timepoint, such as adolescence, interacts with AD-related pathologies to accelerate disease progression later in life. The current study investigates if voluntary exercise in mid-adulthood can recover memory deficits caused by the interactions between adolescence ethanol exposure and AD-transgenes.

**Methods:**

Male and female TgF344-AD and wildtype F344 rats were exposed to an intragastric gavage of water (control) or 5 g/kg of 20% ethanol (adolescent intermittent ethanol; AIE) for a 2 day on/off schedule throughout adolescence (PD27-57). At 6 months old, rats either remained in their home cage (stationary) or were placed in a voluntary wheel running apparatus for 4 weeks and then underwent several behavioral tests. The number of cholinergic neurons in the basal forebrain and measure of neurogenesis in the hippocampus were assessed.

**Results:**

Voluntary wheel running recovers spatial working memory deficits selectively in female TgF344-AD rats exposed to AIE and improves pattern separation impairment seen in control TgF344-AD female rats. There were sex-dependent effects on brain pathology: Exercise improves the integration of recently born neurons in AIE-exposed TgF344-AD female rats. Exercise led to a decrease in amyloid burden in the hippocampus and entorhinal cortex, but only in male AIE-exposed TgF344-AD rats. Although the number of basal forebrain cholinergic neurons was not affected by AD-transgenes in either sex, AIE did reduce the number of basal forebrain cholinergic neurons in female rats.

**Discussion:**

These data provide support that even after symptom onset, AIE and AD related cognitive decline and associated neuropathologies can be rescued with exercise in unique sex-specific ways.

## 1 Introduction

Understanding how alcohol exposure affects the trajectory of healthy and pathological aging is crucial for understanding how lifestyle factors impact the development of dementia and Alzheimer’s disease (AD). Human epidemiological studies suggest that heavy alcohol consumption may lead to earlier onset of AD, especially in individuals with the APOE genetic predisposition for AD (Harwood et al., 2010). Heavy drinking has also been linked to earlier onset of general dementia (Xu et al., 2009; Schwarzinger et al., 2018; Rehm et al., 2019; Lao et al., 2021). Recently, there has been a growing interest in the interactions between chronic alcohol exposure and pathological aging (White et al., 2023). This includes investigating whether early developmental alcohol exposure alters the trajectory of pathological aging. Animal models are being used to directly assess the role of developmental alcohol exposure in the progression to pathological aging. An emerging hypothesis is that the septohippocampal pathway, a critical learning and memory circuit, is especially sensitive to early developmental ethanol exposure and aging (Nunes et al., 2019; Reitz et al., 2021; Deak et al., 2024).

Intermittent alcohol exposure during adolescence has been found to exacerbate age-related spatial memory impairments following an acute ethanol challenge at 18 months of age (Matthews et al., 2017). Another study reported that male, but not female, rats exposed to adolescent intermittent ethanol (AIE) and tested at 19.5 months displayed heightened anxiety and impaired spatial navigation (Matthews et al., 2023). Furthermore, AIE impaired behavioral flexibility following spatial learning in a sex-dependent manner, with again male rats being selectively impaired. However, in a longitudinal design assessing behavior at multiple times across 20 months following AIE, found that spatial memory in the water maze was not affected by AIE, whereas reversal learning was, but advanced age did not worsen the deficit (Matthews et al., 2022).

AIE has also been found to lead to impairment on an object location task that resemble age-related cognitive decline (Reitz et al., 2021). Hippocampal neurogenesis was sensitive to the synergistic interaction between AIE and aging, as young adult AIE rats had a higher number of doublecortin-staining cells than middle-aged AIE rats. However, regardless of age, male AIE rats had lower behaviorally evoked acetylcholine efflux during maze exploration, compared to male control rats, an effect not seen in female rats and an effect not observed when only young males were assessed previously (see Fernandez and Savage, 2017; Kipp et al., 2021). All AIE rats, regardless of age or sex, displayed suppression of the cholinergic neuronal phenotype in the medial septum diagonal band. Thus, these findings support the hypothesis that developmental ethanol exposure leads to accelerated age-related cognitive decline that is associated with alteration in the septohippocampal pathway.

In models of AD, AIE has been shown to accelerate adult brain Aβ_42_ protein levels selectively in females in both the 3xTg-AD mouse model (Barnett et al., 2022) and the APP (amyloid precursor protein)/PSEN (presenilin) mouse model (Ledesma et al., 2021). Our recent data has shown that AIE accelerates impairments in spatial navigation in female TgF344-AD rats and synergistically suppressing hippocampal mature nerve growth factor (mNGF; Reitz et al., 2024). In male rats, AD transgenes alone led to impairments in spatial navigation in early adulthood, which was not further affected by AIE. However, in male rats AIE interacted with AD transgenes to alter pro-NGF, pro-Brain derived neurotrophic factor (BDNF), and p75 neurotrophin receptor leading to suppression of hippocampal vesicular acetylcholine transporter (VAChT). These novel data sets suggest that heavy alcohol exposure during adolescence can synergistically interact with AD transgenes leading to sex-dependent exacerbations of behavioral impairments and changes in neurotrophin markers within the septohippocampal pathway.

Despite its propensity for neurodegeneration, many components of the septohippocampal pathway retain neuroplasticity and the ability for some degree of recovery. Following neurodegenerative events, the septohippocampal pathway is responsive to exercise as a recovery mechanism. In the human population, it is well accepted that regular exercise is associated with reduced incidence and severity of dementia (Abbott et al., 2004; Heyn et al., 2004; Rovio et al., 2005; Larson et al., 2006). The exercised-induced improvements in cognition and brain health are translational across a range of species, including rodents (for review, see da Costa Daniele et al., 2020). Wheel running in rodents consistently leads to cognitive improvements, recovery of synaptic plasticity, increased neurogenesis, and increased clearance of Aβ in models of AD (Adlard et al., 2005; García-Mesa et al., 2011; Revilla et al., 2014; Tapia-Rojas et al., 2016; Lourenco et al., 2019).

Voluntary wheel running exercise is a more effective exercise intervention than forced treadmill exercise to improve spatial learning ability in aging rats, particularly in female aged rats (Belviranli and Okudan, 2022). In rodent models of natural age-related cognitive decline, voluntary wheel running for as short as three weeks revitalized neurogenesis through adult-born granule cells (Trinchero et al., 2019). Voluntary wheel running for the same period of time (three weeks) in aged Tg2576 mice decreased Aβ, altered proinflammatory markers, and improved cognitive performance (Nichol et al., 2007; Parachikova et al., 2008). A study investigating the effects of exercise in male TgF344-AD rats, long-term treadmill exercise (3×/week for 8 months) reduced anxiety and depressive-like phenotypes at 12- and 18-months of age (Wu et al., 2020; Yang et al., 2022). Long-term exercise attenuated AD-related memory impairments on the Barne’s maze, passive avoidance task, and cued fear conditioning at 18-months of age in male TgF344-AD rats (Yang et al., 2022). By 12 months old, this exercise paradigm led to an attenuation of Aβ deposition, tau hyperphosphorylation, microgliosis, and oxidative damage (Wu et al., 2020), a finding that was replicated with 18-month old rats (Yang et al., 2022). It has been suggested that exercise attenuates cognitive impairment in an amyloid-independent way— by decreasing microglial activation and increasing BDNF in the hippocampus and cerebral cortex (Xiong et al., 2015).

In addition, voluntary wheel running has been shown to effectively prevent the loss of neurogenesis in the HPC and recovers the cholinergic neuronal phenotype in the basal forebrain following AIE (Vetreno and Crews, 2018; Vetreno et al., 2018, 2020). However, studies investigating the effect of exercise on the synergistic deficits following AIE in AD rodent models has done been widely reported. Voluntary wheel running has not yet been studied as a measure to prevent *or* recover the cognitive decline, Aβ burden, tauopathy, or loss of cholinergic tone and neurogenesis seen in the TgF344-AD model, with or without early-life exposure to ethanol—particularly in female rats. The current study was designed to determine whether voluntary wheel running could recover spatial memory dysfunction, cholinergic neural phenotype with the medial septal/diagonal band, hippocampal neurogenesis, and plaque burden in the dorsal hippocampus and entorhinal cortex in both male and female wild-type and AD rats at 7 months of age, some of which underwent AIE procedures. Given prior findings, we hypothesized that female AD rats would be the most sensitive to AIE, but display robust behavioral and neural recovery with exercise.

## 2 Materials and methods

### 2.1 Subjects

Male (*N* = 75) and female (*N* = 75) Fischer 344 rats were bred at Binghamton University using female wild-type F344 rats (obtained from Charles River, Stoneridge, NY, USA) and male TgF344-AD+ rats (AD; obtained from RRRC, Columbia, MO, USA). Of the 150 total rats, 80 were AD (Cohen et al., 2013) and 70 were their wild-type littermates. On postnatal day (P) 21, all rats were weaned from the dam and genotyped using ear-punched tissue. Tissue was sent to Transnetyx (Cordova, TN, USA) for automated genotyping. All rats ranged from P27-29 at the start of ethanol exposure (see below). Only one rat per sex per litter was randomly assigned to a given exposure group. Rats were pair-housed post-weaning and given free access to food and water. All rats were housed in a temperature-controlled colony room that maintains a 12-h light/dark cycle from 7:00 AM to 7:00 PM. Rats were given wood chew blocks and crinkle paper as enrichment and were housed in standard bedding. All procedures and housing are in accordance with the Institutional Animal Care and Use Committee at Binghamton University.

### 2.2 Adolescent intermittent ethanol exposure

**FIGURE 1:**
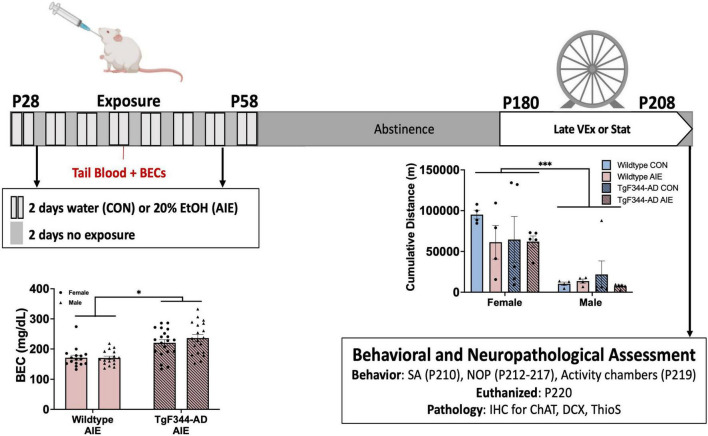
Timeline for Experimental procedures. All rats underwent a gavage procedure of 20% ethanol (AIE) or tap water (CON) for two days on/two days off from postnatal day (P) 28–58. Blood ethanol concentrations (BECs) exceeded binge levels (> 80 mg/dL). BECs were greater for TgF344-AD rats than wild-type rats. All rats remained in abstinence until the onset of voluntary wheel running exercise at 6-months of age, in which half of the rats in each condition were placed into a home cage with open access to a running wheel 24 h a day for 28 days. Females, regard less of genotype or treatment ran more than males. All rats were then tested on spontaneous alternation (SA), novel object in place (NOP), and activity at 7-months old. All rats were euthanized within 2 weeks of the termination of the exercise paradigm, and tissue was processed for IHC. *p < 0.05; ***p < 0.001.

Rats of both sexes were exposed to either a binge-type forced gavage ethanol exposure (AIE: *N* = 74) or tap water gavage control exposure (CON: *N* = 76). Rats were exposed to 16 intragastric gavages of either 20% ethanol (*v/v*) administered at a dose of 5 g/kg, or an equal volume of water (see Figure 1). The exposure cycle followed a two-day on/off cycle from P28-58. Tail blood samples were collected one hour after the eighth exposure (P41) to ensure binge levels of ethanol consumption (BEC > 0.08) were met. All blood samples were processed using an AM1 Alcohol Analyzer (ANALOX Instruments, England) and the blood ethanol content (BEC) of each animal was recorded. Following the final ethanol or water exposure, all rats remained in an abstinence (no ethanol) phase for the remaining duration of the study.

#### 2.2.1 Exercise paradigm

Rats of both sexes, genotypes, and exposure conditions were randomly assigned to one of two exercise conditions: (1) a stationary control group (*n* = 72) or (2) a voluntary exercise (VEx) group (*n* = 72; see Figure 1 for timeline). The rats in the VEx group remained in their home cage until they underwent the exercise paradigm at 6 months of age (28 days; P180-208), 122 days following the conclusion of AIE or control treatment. At the end of the 4-week exercise paradigm, rats were returned to their home cage until behavioral testing. Rats in the stationary control group remained pair-housed in their home cages until behavioral testing.

Rats in the VEx group also remained pair-housed and were placed into standard housing cages (48.3 × 26.7 × 20.3 cm) with free 24-h access to a running wheel attachment (Lafayette Instrument Company, Lafayette, IN) for 4 weeks. Daily running (cumulative m/day) was recorded via AWM^®^ software (Lafayette Instrument Company) of wheel revolution counts. Rats were pair-housed while in the exercise apparatus as previous data indicate that social isolation not only leads to a decrease in wheel running, but also impedes exercise-induced hippocampal neurogenesis (Stranahan et al., 2006; Leasure and Decker, 2009). Therefore, the cumulative meters per day were recorded and analyzed by cage (two rats per cage). Cage mates were always of the same Sex, Genotype, and Exposure group.

### 2.3 Behavioral testing at 6-months old

Beginning at P210, rats in both the stationary control group and the VEx group underwent behavioral testing. All behavioral testing was completed within 12 days. Brains were collected at P224—exactly 14 days following the last day of exercise.

#### 2.3.1 Spontaneous alternation

Before testing, all rats underwent food restriction to maintain their weight at 85–90% of their normal feeding weight. Additionally, they were handled for seven days. The test for spontaneous alternation was conducted in an elevated plus maze measuring 105.5 cm × 14.4 cm × 15 cm, with transparent exterior walls. Spatial cues were prominently displayed on the walls to guide the rats through the maze, and these cues remained consistent for each rat.

Rats were individually placed in the testing room and allowed to acclimate for 30 min before the test began. During testing, each rat was placed in the center of the maze and given 18 min to freely explore. The number of unique arm entries, defined as all four paws being in an arm, was recorded. A complete spontaneous alternation was counted when a rat made four consecutive unique arm entries. Performance was measured as the percentage of correct alternations, calculated as the ratio of actual alternations to possible alternations (total alternations/[total arm entries-3]) multiplied by 100. Rats must make at least 13 arm entries to meet task completion criterion. After the test, the rats were returned to their home cages and provided with free access to food to regain their pre-restriction weights.

#### 2.3.2 Novel object in place

Rats underwent 48 h of unrestricted feeding and recovery between spontaneous alternation testing and the commencement of novel object in place (NOP) testing. The parameters for NOP testing were established in prior studies (Cai et al., 2012; Reitz et al., 2021). The testing occurred in a plexiglass open-field arena (38.5 cm × 70 cm × 36 cm) with a black floor and white walls. For the placement recognition testing, identical square-shaped plastic blocks (6.5 cm × 6.5 cm × 2.5 cm) with 5 holes were utilized. Each object was affixed to the arena using Velcro and cleaned between trials with a diluted antibacterial solution. The encoding and retention test trials were video recorded and analyzed by experimenters who were blind to the experimental conditions. These experimenters adhered to a detailed protocol and underwent extensive training to ensure strong inter-rater reliability.

Habituation: Rats were placed in the empty arena (without objects) for 10 min on two consecutive days and allowed to explore freely. The habituation phase was not recorded.

Encoding: Both objects were positioned in the arena (11.1 cm × 15.28 cm from the nearest walls; see Figure 3). Approximately 24 h after the final habituation session, rats were placed in the arena and permitted to explore freely for 6 min. Two encoding sessions took place 24 h apart. The exploration of the objects was recorded and analyzed to establish a baseline for the exploration of each object.

**FIGURE 2:**
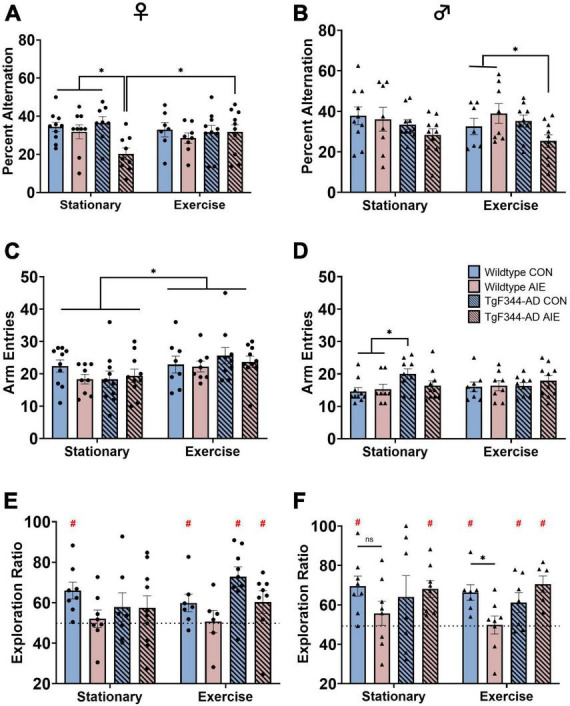
Spatial behaviors: Spontaneous alternation scores for both female **(A)** and male **(B)** rats. Arm entries on the spontaneous alternation task for female rats **(C)** and male rats **(D)**. Circle data points represent female rats; triangle data points represent male rats. AD-AIE female rats had lower alternation scores, which was corrected by exercise. In contrast, in male AD-AIE rats exercise decreased alternation scores. Novel object in place scores for both female **(E)** and male **(F)** rats. Dotted line represents chance (50) level. For female rats in the stationary condition, only WT-CON rats performed above chance. In contrast, in the exercise condition, WT-CON, AD-CON and AD-AIE female rats performed above chance level. In the stationary condition, male WT-CON, and AD-AIE rats performed above chance level; whereas in the exercise condition, male WT-CON, AD-CON and AD-AIE rats performed above chance levels. *p < 0.05; #significant difference from a theoretical “chance” value of 50.

**FIGURE 3:**
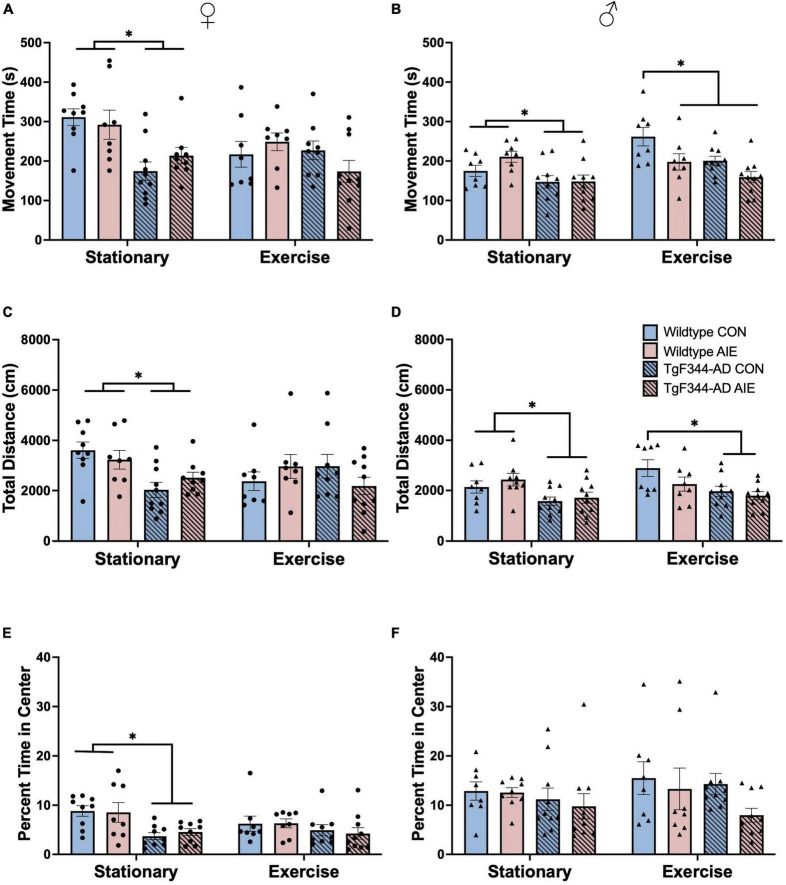
Analyses of locomotor activity from activity chambers at 7-months of age for female rats **(A,C,E)** and male rats **(B,D,F)**. **(A)** total movement time (s; out of 1,800 s) for female rats, **(B)** total movement time (s; out of 1,800 s) for male rats, **(C)** total distance moved (cm) for female rats, **(D)** total distance moved (cm) for male rats, **(E)** percentage of time in the center of the arena for female rats, and **(F)** percentage of time in the center of the arena for male rats. In the stationary condition, WT AD rats, regardless of Sex were more active, as determined by movement time and total distance, than AD rats. In male rats, exercise decreased activity as a function of AIE and AD transgenes. In the stationary condition, AD transgenes, selectively in females, decreased time spent in the center of the arena. *p < 0.05.

Retention: One object remained in its original location (familiar), while the other object was moved slightly adjacent to the first object (novel; 15.4 cm × 35.25 cm from its nearest walls; see Figure 3). Approximately 24 h after the second encoding trial, rats were placed in the rearranged arena for 6 min and allowed to explore freely. The exploration of each object was recorded and analyzed to determine differences in exploration time between the two objects. An exploration ratio was calculated for each rat by dividing the time spent exploring the object in the novel location by the total time spent exploring each object in the retention session. An exploration ratio of 0.5 would indicate equal time spent with each object, suggesting that the rat does not recognize a change in location. A ratio greater than 0.5 would indicate more time spent with the object in the novel location, suggesting that the rat retained the memory of the previous locations and recognized that one of the objects had moved. To meet task completion criterion, rats must make contact with each object at least once and explore each for a minimum of 1.0 s to ensure acknowledgment of both objects. Due to the potentially heightened levels of anxiety in this model, many rats did not explore the objects adequately enough to meet task criterion. After completing the NOP testing, the rats were returned to their home cages.

#### 2.3.3 Activity chambers

After completing the NOR testing at around 6.5 months of age, we assessed any changes in generalized locomotor activity due to genotype or ethanol exposure using activity chambers from Accuscan Instruments (Columbus, OH, USA). Each of the six acrylic chambers measured 41 cm × 41 cm × 30.5 cm and was surrounded by a 15 × 15 infrared photocell array. These chambers were synchronized with Versamax and Versadat software (Accuscan Instruments) to measure and record patterns of horizontal and vertical beam breaks. For a period of 30 min, the total time spent moving (in seconds), time spent in the margins of the chamber (in seconds), and time spent in the center of the chamber (in seconds) were recorded every 5 min.

### 2.4 Tissue preparation

Two days following completion of behavioral testing, rats were euthanized with Fatal-Plus (Vortech Pharmaceuticals, Dearborn, MI, USA) and perfused (Masterflex Easy-Load Console Drive; 7518-00; Cole Palmer Instrument Co, Vernon Hills, IL, USA) with ice-cold phosphate-buffered saline and 4% paraformaldehyde in 0.1M phosphate buffer (pH = 7.2). Brains were post-fixed at 4° overnight in 4% PFA and then transferred to a 30% sucrose solution (in 0.1M PBS) at 4° until slicing. Whole brains were sliced horizontally at 40 μm using a sliding microtome (Sm2000r Leica Biosystems, Wetzler, Germany). All tissue slices were kept at −20° in an antifreeze solution (62.8 mg NaH_2_PO_4_, 2.18 g Na_2_HPO_4_, 160 mL dH_2_O, 120 mL ethylene glycol and 120 mL glycerol) until immunohistochemistry staining.

### 2.5 Immunohistochemistry

#### 2.5.1 Choline acetyltransferase (ChAT)

Every 7th horizontal section (6 sections per rat) of the MS/DB underwent immunohistochemical staining for ChAT+ expression. Sections were processed using well plates that allow for the free-floating of the tissue. Phosphate buffered saline (PBS; 0.1M; pH = 7.4) was used to wash the tissue of antifreeze solution. Sections were then quenched in a 0.6% hydrogen peroxide solution for 30 min. Tissue sections were blocked in a solution of 4% rabbit serum (S-5000; Vector Laboratories) and 0.1% Triton X-100 (X-100; Millipore Sigma; Burlingame, MA) in a 0.1M PBS solution. Tissue sections were then immediately transferred to a ChAT primary antibody (AB144P; EMD Millipore; Billerica, MA, USA; 1:200 dilution) made in blocking solution, where they incubated overnight at 4°C. The following day, tissue sections underwent another PBS wash and were then placed into a secondary antibody (biotinylated rabbit anti-goat IgG; BA-5000; Vector Laboratories; Burlingame, CA, USA; 1:200 dilution) made in blocking solution for two hours. After another PBS rinse, the tissue sections were incubated in an avidin/biotin complex (VECTASTAIN Elite ABC HRP Kit, Vector Laboratories) diluted in 0.1M PBS for 1 h. Sections were washed in PBS and then developed using SIGMA*FAST* 3,3′-Diaminobenzidine (DAB) tablets (D4418; Millipore Sigma; Burlington, MA, USA) dissolved in 0.1M PBS. Following the development of the ChAT stain, tissue was rinsed for a final time in 0.1M PBS. Sections were then mounted, and cover slipped using VectaMount permanent mounting medium (H-5000; Vector Laboratories).

#### 2.5.2 Doublecortin (DCX)

Every 8th horizontal section (3 sections per rat) of the dorsal hippocampus and every 8th section (3 sections per rat) of the ventral hippocampus were pulled for processing of DCX+ expression in the granule cell layer of the dentate gyrus. Free-floating sections were washed using Tris-buffered saline (TBS; 0.1M; pH = 7.4). Sections were quenched using a 0.6% hydrogen peroxide solution for 30 min, which was followed by another TBS wash. Tissue slices were then blocked using a solution consisting of 4% rabbit serum (S-5000; Vector Laboratories) and 0.1% Triton X-100 (X-100; Millipore Sigma; Burlingame, MA) in 0.1M TBS. Tissue was then immediately transferred to an overnight incubation (at 4°C) in primary antibody (goat anti-DCX; SC-8066; Santa Cruz; Dallas, TX; 1:200 dilution) made in blocking solution. The following day, tissue was washed in 0.1 TBS and then incubated in a secondary antibody (biotinylated anti-goat IgG; BA-5000; Vector Laboratories; 1:200 dilution) made with blocking solution for two hours. Tissue went through another TBS wash followed by an incubation in an avidin/biotin complex (VECTASTAIN Elite ABC HRP Kit (PK-6100), Vector Laboratories) in 0.1M TBS for one hour. Tissue was washed in TBS and then developed using SIGMA*FAST* DAB tablets with Metal Enhancer (D0426; Millipore Sigma) dissolved in 0.1M TBS. After a final wash in 0.1M TBS, sections were mounted and then cover slipped using VectaMount permanent mounting medium (H-5000; Vector Laboratories).

#### 2.5.3 Thioflavin-S staining for amyloid burden

Sections from the hippocampus and entorhinal cortex were stained for beta amyloid plaque formation using Thioflavin-S (ThioS; Cohen et al., 2013). Every 12th horizontal section with both the hippocampus and entorhinal cortex (6 sections per rat) were pulled for Thioflavin-S (ThioS) staining to visualize and analyze amyloid beta plaque formation. Tissue sections were washed in 1X PBS, mounted onto clean gelatin-coated slides, and allowed to dry flat for 24 h. Slides were then placed through another round of PBS washes, followed by a 20-min incubation in a 0.05% potassium permanganate solution (made with 1X PBS). Tissue sections were washed in PBS and then immersed in a 0.2% potassium metabisulfite and 0.2% oxalic acid solution (made with 1X PBS) for 2 min. Following another wash in PBS, the slides were immersed in a 0.0125% ThioS and 40% ethanol solution for 3 min. Slides were then placed in 50% ethanol for 15 min, followed by a final PBS wash. Slides were kept wet and coverslipped with Prolong Diamond Antifade Mountant (P36965; ThermoFisher Scientific, Waltham, MA, USA).

#### 2.5.4 Cell quantification of MS/DB, dorsal HPC, and ERC

Images were captured using a SLIDEVIEW VS200 Research Slide Scanner (OLYMPUS Life Sciences; Waltham, MA) with a 40X objective. Image analysis and blind manual cell counting was completed using the OlyVIA plugin for ImageJ. One hemisphere of each tissue section was quantified for immunopositivity (ChAT+, DCX+, ThioS) in their respective brain regions. ChAT+ cells in the MS/DB and DCX+ cells in the hippocampus were manually counted for each section of tissue. Area fraction (percentage of the ROI covered) of DCX in the hippocampus and ThioS in the HPC and ERC was calculated using ImageJ. Total number of plaques and average plaque size in the hippocampus and entorhinal cortex were automatically quantified using ImageJ. Threshold limits and ROI sizes were manually calculated and made consistent across all animals.

### 2.6 Statistical analyses

Analyses were performed in GraphPad Prism (version 9.5.1). A univariate factorial ANOVA (Exposure [AIE, Control] x Exercise [VEx, Stationary] x Sex [Female, Male]) found that female rats (M = 35,398.93, SD = 6,384.57) ran substantially more cumulative meters than male rats (M = 6,680.66, SD = 3,071.55; *F*[1, 64] = 64.34, *p* < 0.0001). Therefore, all subsequent behavioral and histological analyses were separated by sex.

Univariate factorial ANOVAs (separated by sex; Exposure [AIE, Control] x Exercise [VEx, Stationary], Genotype [AD, Wild-type]) were used to analyze spontaneous alternation scores, arm entries, and NOP measures. Paired *t*-tests were used to determine differences in NOP exploration ratios from a theoretical value of 0.50 (equal time spent with each object) for each group (Mathiasen and DiCamillo, 2010; Reitz et al., 2021). Univariate factorial ANOVAs (separated by sex; Exposure x Exercise, Genotype were used to examine ChAT+ (total count) and DCX+ (total count, area fraction) measures in their respective brain regions. For AD rats only, individual univariate factorial ANOVAs (separated by sex; Exposure x Exercise [) were used to analyze ThioS+ measures (total count, average size, and area fraction) in the hippocampus and entorhinal cortex separately. When appropriate, Fisher’s LSD test was used for post-hoc analyses. Data points two standard deviations above or below the initial group mean were removed as outliers. Pearson correlations were used to assess linear relationships between performance on behavioral tasks and DCX+ and ThioS expression in corresponding brain regions.

## 3 Results

Recovery in behavior or pathology due to voluntary wheel running exercise is defined as a group’s return to baseline levels following the exercise paradigm; baselines are defined by the wild-type water control group in each exercise condition.

### 3.1 BECs

AD rats had higher BECs (M = 220.4 mg/dL) than wild-type rats (M = 171.1 mg/dL) (effect of Genotype; *F*[1, 68] = 33.93, *p* < 0.001; see Figure 1). Sex of the rat did not influence BEC levels (*p* = 0.44).

### 3.2 Voluntary wheel running exercise

Female rats ran more cumulative meters than male rats across a 28-day voluntary wheel running paradigm (effect of sex; *F*[1, 28] = 29.39, *p* < 0.0001; see Figure 1). There were no significant group differences based on Exposure or Genotype in male or female rats.

### 3.3 Spontaneous alternation

For female rats, data points from four rats were excluded (one outlier, three failures to meet task criterion). Final n’s include: wild-type stationary (10 water control, 9 AIE), wild-type exercise (7 water control, 8 AIE), AD stationary (8 water control, 9 AIE), AD exercise (10 water control, 10 AIE). For male rats, data points from three rats were excluded (one outlier, two failures to meet task criterion). Final n’s include: wild-type stationary (10 water control, 8 AIE), wild-type exercise (7 water control, 8 AIE), AD stationary (10 water control, 10 AIE), AD exercise (9 water control, 10 AIE).

Stationary female AD rats exposed to AIE were impaired on spontaneous alternation, a task of spatial working memory dependent on the dorsal hippocampus (see Figures 2A,B). There was an overall Exposure effect with female rats exposed to AIE performing worse than water control female rats (Figure 2A), regardless of Genotype or Exercise condition (*F*[1, 63] = 5.88, *p* = 0.018). Female AD AIE rats had significantly lower percentages of correct alternations compared to all other female groups (wild-type water [*p* = 0.003], wild-type AIE [*p* = 0.02] and AD water [*p* = 0.001]), when in the stationary condition. A voluntary wheel running exercise paradigm at 6 months of age recovered the impairment of spatial working memory in the AD AIE exposed female rats (*p* = 0.015). Exercise increased the total number of arm entries in female rats (effect of Exercise; *F*[1, 67] = 7.01, *p* = 0.01; see Figure 2C). The total number of arm entries did not correlate with the percent alternation in female rats (*p* = 0.37).

Male AD rats performed worse than male wild-type rats on spontaneous alternation (effect of Genotype: *F*[1, 64] = 4.41, *p* = 0.04; see Figure 2B). Voluntary exercise in AD male rats exposed to AIE resulted in an impairment in spontaneous alternation compared to wild-type male rats exposed to AIE who had also undergone voluntary exercise (*p* = 0.02). There was no improvement in percent alternations due to voluntary wheel running in the AD male rats exposed to AIE (*p* = 0.55). AD male rats made more arm entries during spontaneous alternation than wild-type male rats (see Figure 2D; effect of genotype; *F*[1, 64] = 4.25, *p* = 0.043); specifically, in the stationary group, AD water control rats made more arm entries than wild-type water control rats (*p* = 0.008) and wild-type AIE exposed rats (*p* = 0.026). There was no main effect of Exposure or Exercise on arm entries in male rats (*p*’s > 0.81). The total number of arm entries did not correlate with percent alternation in male rats (*p* = 0.56).

### 3.4 Novel object in place

For female rats, data points from 13 rats were excluded for failure to meet exploration criterion for the task. Final n’s include: wild-type stationary (8 water control, 8 AIE), wild-type exercise (7 water control, 6 AIE), AD stationary (7 water control, 10 AIE), AD exercise (8 water control, 8 AIE). For male rats, data points from 16 rats were excluded (one outlier, 15 failures to meet exploration criterion). Final n’s include: wild-type stationary (8 water control, 8 AIE), wild-type exercise (7 water control, 8 AIE), AD stationary (7 water control, 8 AIE), AD exercise (7 water control, 6 AIE).

#### 3.4.1 Group differences

Female rats exposed to AIE had lower exploration ratios than water-control female rats (effect of Exposure; *F*[1, 55] = 5.48, *p* = 0.023; see Figure 2E). There was no effect of Exercise or Genotype on NOP performance in female rats (*p*’s > 0.21).

There was a significant Genotype X Exposure interaction (*F*[1, 51] = 6.87, *p* = 0.011) in male rats on NOP performance (see Figure 2F): Exposure to AIE impaired performance more in the wild-type male rats compared to the AD male rats. AIE-exposed wild-type male rats performed significantly worse than water control wild-type rats following voluntary wheel running exercise (*p* = 0.05), an effect that was not seen in the stationary groups (*p* = 0.08).

#### 3.4.2 Difference from chance performance

Exploration ratios above 50 (as determined by *t*-tests) indicate more time spent with the object in the novel location, which is interpreted as intact memory for the familiar location as rats tend to spend more time exploring novelty (Ennaceur and Delacour, 1988). When exploration ratios are not significantly above 50, this implies equal time with the objects in the novel and the familiar locations. This is interpreted as impaired pattern separation ability, or also considered discriminating novel locations at “chance level.”

In the stationary condition, only wild-type control female rats performed significantly above chance on NOP (*t*-test; *p* = 0.002). All other stationary female groups performed at chance level (all *p*’s > 0.06). Following voluntary wheel running, wild-type water control (*p* = 0.043), AD water control (*p* < 0.001), and AD AIE exposed (*p* < 0.001) female rats all performed above chance level. Wild-type AIE exposed female rats remained at chance level following exercise (*p* = 0.23).

In the stationary condition, only wild-type water controls (*p* = 0.001) and AD AIE exposed (*p* < 0.001) male rats performed significantly above chance. Following voluntary wheel running, wild-type water control (*p* < 0.001), AD water control (*p* = 0.035), and AD AIE exposed (*p* < 0.001) male rats all performed above chance level.

There was no correlation between total amount of time spent exploring the objects and exploration ratios.

### 3.5 Activity chambers

Movement time: AD female rats spent less total time moving in the activity chambers compared to wild-type female rats (effect of Genotype; *F*[1, 63] = 14.16, *p* < 0.001; see Figure 3A). There was an Exercise X Genotype interaction for female rats (*F*[1, 63] = 4.05, *p* = 0.048) and a trend toward an Exercise X Genotype X Exposure interaction (*F*[1, 63] = 3.72, *p* = 0.058).

There was an effect of Genotype in male rats, with AD rats having lower movement times than wild-type rats (*F*[1, 64] = 16.67, *p* < 0.001; see Figure 3B). There was an Exercise X Exposure interaction for male rats (*F*[1, 64] = 9.30, *p* = 0.003). Male rats that underwent voluntary exercise spent more time moving in the activity chambers than stationary male rats (*F*[1, 63] = 8.83, *p* = 0.004).

Total distance (cm): AD female rats moved less in the activity chambers compared to wild-type female rats (effect of Genotype; *F*[1, 63] = 5.73, *p* = 0.01; see Figure 3C). There was an Exercise X Genotype interaction for female rats (*F*[1, 63] = 4.21, *p* = 0.044): Exercise resulted in a decrease in movement in wild-type water controls (*p* = 0.022), but a trend toward increased movement in AD water controls (*p* = 0.06). There was an Exercise X Genotype X Exposure interaction (*F*[1, 63] = 4.649, *p* = 0.035) on distance moved for female rats: exercise seemed to decrease total distance moved in wild-type water control rats, but increased total distance moved in AD water controls. Exercise did not appear to influence total distance moved in AIE-exposed female rats of either genotype.

There was an effect of Genotype in male rats, with AD rats moving less in the activity chambers than wild-type rats (*F*[1, 65] = 16.82, *p* = 0.0001; see Figure 3D). There was a trend toward a significant Exercise X Exposure interaction in male rats (*F*[1, 65] = 3.67, *p* = 0.059). In male rats, exercise resulted in an increase in distance moved only in wild-type water control males (*p* = 0.033). There was no main effect of Exercise or Exposure on total distance moved in male rats (*p*’s > 0.11).

Time in center (%): The percentage of time spent in the center of the activity chamber was calculated by dividing time spent in the center by total time in the chamber (1800s). AD female rats had a higher center-to-margin ratio than wild-type female rats, indicating that they spent more time in the margins for every second they spent in the center of the activity chambers (Effect of Genotype: *F*[1, 62] = 12.78; *p* < 0.001; see Figure 3E). AD male rats also had a higher center-to-margin ratio than wild-type male rats (Effect of Genotype: *F*[1, 63] = 5.09; *p* = 0.028; see Figure 3F). AIE-exposed male rats had a higher center-to-margin ratio than water control male rats (effect of Exposure; *F*[1, 63] = 6.85; *p* = 0.011). There was a trend toward an Exercise X Genotype interaction for center-to-margin ratios in male rats (*F*[1, 63] = 3.77, *p* = 0.056), with voluntary exercise decreasing the center-to-margin ratio for AD water control rats back to wild-type control levels. The center-to-margin ratio for AD AIE exposed male rats remained heightened following exercise (*p* = 0.044).

### 3.6 ChAT immunohistochemistry

**FIGURE 4:**
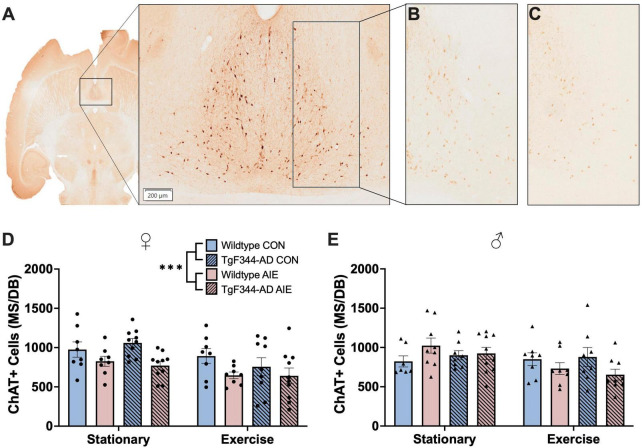
**(C)** Image of ChAT+ cells in the MS/DB in a horizontal slice (Interaural 3.40 mm; Bregma –6.60 mm) of a 7-month-old F344 rat **(A)**. ChAT+ cells in the MS/DB of female wild type control stationary condition rat **(B)** and a female TgF344-AD AIE stationary condition rat **(C)** demonstrating the effect of AIE on loss of ChAT+ cells in female rats. In female rats AIE suppressed the number of ChAT neurons **(D)**, an effect not seen in male rats **(E)**. ***p < 0.001.

Data points for four male rats were excluded for poor tissue/image quality. Final male n’s include: wild-type stationary (8 water control, 9 AIE), wild-type exercise (8 water control, 8 AIE), AD stationary (8 water control, 10 AIE), AD exercise (8 water control, 10 AIE). A sample of the region and ChAT+ cells is shown in Figures 4A–C.

Exposure to AIE resulted in a significant decrease in ChAT+ cells in the MS/DB in female rats, regardless of Genotype or Exercise condition (effect of Exposure; *F*[1, 63] = 11.81, *p* = 0.001; see Figure 4D). Interestingly, voluntary wheel running exercise also resulted in a decrease of ChAT+ cells in the MS/DB of female rats (effect of Exercise; *F*[1, 63] = 8.940, *p* = 0.004). Both wild-type (*p* = 0.048) and AD (*p* = 0.03) female rats exposed to AIE had fewer ChAT+ cells compared to wild-type water controls following voluntary wheel running. There was no main effect of Genotype on ChAT+ cells in the MS/DB of female rats (*p* = 0.63).

Voluntary wheel running resulted in a decrease of ChAT+ cells in the MS/DB of male rats (effect of Exercise; *F*[1, 59] = 5.50, *p* = 0.02; see Figure 4E). There was also an Exercise X Exposure interaction in male rats (*F*[1, 59] = 5.74, *p* = 0.02); both wild-type and AD AIE exposed male rats show a decrease in ChAT+ cells following voluntary exercise, whereas water controls of both genotypes showed no change. There was no main effect of Genotype or Exposure on ChAT+ cells in the MS/DB of male rats (*p*’s > 0.61).

### 3.7 DCX immunohistochemistry

**FIGURE 5:**
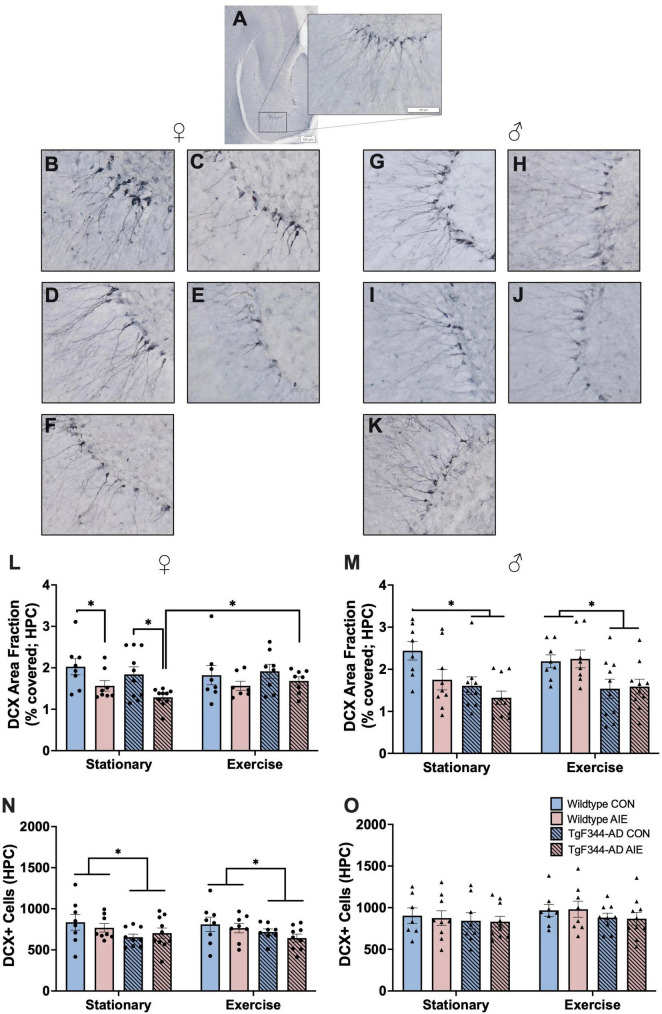
DCX+ cells in the subgranular zone and molecular layer areas of the hippocampus (HPC). **(A)** Image of DCX+ staining in the HPC of a horizontal slice (Interaural 3.90 mm; Bregma –6.10 mm) of a 7-month-old F344 rat. In female rats, images of: **(B)** Wild-type water control stationary, **(C)** Wild-type AIE-exposed stationary, **(D)** TgF344-AD water control stationary, **(E)** TgF344-AD AIE-exposed stationary, **(F)** TgF344-AD AIE-exposed following exercise. In male rats images of: **(G)** Wild-type water control stationary, **(H)** Wild-type AIE-exposed stationary, **(I)** TgF344-AD water control stationary, **(J)** TgF344-AD AIE-exposed stationary, **(K)** TgF344-AD AIE-exposed following exercise. Percentage of the HPC covered by DCX+ cell bodies and processes (area fraction) were analyzed in both female rats **(L)** and male rats **(M)**. In female rats AIE decreased the area covered by DCX+ neurons, which was corrected by exercise. In male rats AD transgenes decreased the area covered by DCX+ neurons. Total count of the number of DCX+ cell bodies in the HPC was analyzed in both female **(N)** and male **(O)** rats. In female rats, AD transgenes decreased the number of DCX+ cells. *p < 0.05.

Data points for three female rats were excluded for poor tissue/image quality. Final n’s female include: wild-type stationary (8 water control, 8 AIE), wild-type exercise (8 water control, 8 AIE), AD stationary (10 water control, 10 AIE), TgF344-AD exercise (10 water control, 9 AIE). See Figures 5A–K for images of DCX staining in the subgranular zone of the hippocampus and entorhinal cortex.

Percent of Subgranular Zone and Molecular Layer Areas Occupied by DCX— Female rats exposed to AIE had less area covered by DCX+ neurons and their processes in the dentate gyrus compared to water-control female rats (effect of Ethanol exposure; *F*[1, 59] = 11.37, *p* = 0.001; see Figure 5L). This deficit was not found in rats that underwent voluntary wheel running exercise (when compared to water controls; all *p*’s > 0.28). There was no main effect of Exercise or Genotype on area covered by DCX+ neurons in the DG of female rats (*p*’s > 0.55). There were no significant interactions between Exercise, Genotype, or Exposure (all *p*’s > 0.14).

Male AD rats had less area covered by DCX+ neurons in the DG than male wild-type rats (effect of Genotype; *F*[1, 64] = 19.94, *p* < 0.001; see Figure 5M). There was a trend toward a significant Exercise X Exposure interaction (*F*[1, 64] = 3.517, *p* = 0.065). In the stationary male groups, wild-type AIE (*p* = 0.02), AD water controls (*p* = 0.007), and TgF344-AD AIE (*p* < 0.001) rats had less area covered by DCX+ neurons compared to wild-type water controls. When rats underwent voluntary wheel running exercise, AIE-exposed wild-type rats maintained comparable levels of DCX+ area coverage to water wild-type controls (*p* = 0.85). However, AD rats in both water control (*p* = 0.02) and AIE-exposed (*p* = 0.04) groups still exhibited a loss of DCX+ area coverage when compared to water wild-type controls. There was no main effect of Exercise or Exposure on area covered by DCX+ neurons in the DG of male rats (*p*’s > 0.13).

Total DCX+ cell count—AD female rats had less total DCX+ neurons in the GCL of the DG than wild-type female rats (effect of Genotype; *F*[1, 60] = 6.886, *p* = 0.011; see Figure 5N). There was no effects of Exposure or Exercise on total DCX+ neurons in female rats (*p*’s > 0.43).

There was no effect of Genotype, Exposure, or Exercise on total DCX+ neurons in the GCL of the DG in male rats (all *p*’s > 0.18; see Figure 5O).

### 3.8 ThioS staining

**FIGURE 6:**
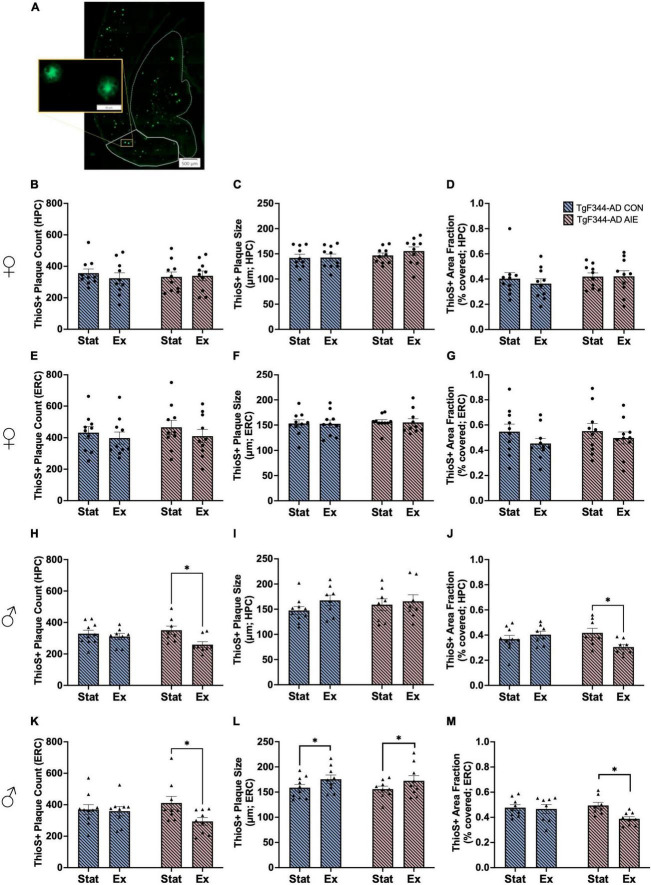
Image of horizontal orientation of the brain for Thioflavin-S (ThioS) staining of amyloid beta plaques in the entorhinal cortex (ERC, solid white outline) and hippocampus (HPC, dashed white outline) of a 7-month-old TgF344-AD rat **(A)**. Analyses of ThioS+ amyloid plaques in the hippocampus (HPC; **B–D**) and entorhinal cortex (ERC; **E–G**) of female TgF344-AD rats, in both Stationary (Stat) and Exercise (Ex) groups. Analyses of ThioS+ amyloid plaques in the hippocampus (HPC; **H–J**) and entorhinal cortex (ERC; **K–M**) of male TgF344-AD rats, in both Stationary (Stat) and Exercise (Ex) groups. Exercise, selectively in male AD rats, decreased plaque count and area covered by plaques in both the HPC and EC. In addition, exercise selective increased plaque size in the EC. *p < 0.05.

Previous studies have found no Aβ_40_ positive plaques in the brains of wild-type F344 rats (Tournier et al., 2021). Studies selectively examine plaque density in AD rats as a function of age or treatment and do not include wild-type rats (Rorabaugh et al., 2017; Morrone et al., 2020; Saré et al., 2020; Ceyzériat et al., 2021). We examined the brain of 16 wild-type F344 rats and observed no ThioS-positive plaques in their brains, regardless of Sex, Ethanol exposure, or Exercise treatments. Thus, the analysis of ThioS+ plaques was only performed in AD rats (male and female separately) as a function of ethanol exposure and exercise (see Figure 6A for image of ThioS staining).

There was no effect of Exposure or Exercise on plaque size, plaque count, or percent of area covered in the hippocampus or entorhinal cortex of female AD rats (all *p*’s > 0.21; see Figures 6B–G).

Male AD rats who underwent voluntary wheel running had fewer plaques in the HPC than male AD rats that remained stationary (effect of Exercise; *F*[1, 30] = 6.24, *p* = 0.018; see Figure 6H). Specifically, voluntary exercise reduced the number of plaques in the hippocampus of AD male rats that underwent AIE, compared to stationary AIE males (*p* = 0.015). There was no main effect of Exposure on plaque count in the hippocampus of male rats (*p* = 0.51). There was no effect of Exercise or Exposure on plaque size in the hippocampus of male rats (all *p*’s > 0.22; see Figure 6I). There was an interaction between Exercise X Exposure for area fraction in the hippocampus of male AD rats *F*[1, 30] = 6.24, *p* = 0.017; see Figure 6J). Voluntary exercise reduced percent of area covered by plaques in the hippocampus of AD male rats who were exposed to AIE, compared to stationary AIE males (*p* = 0.025).

Voluntary exercise reduced the number of plaques in the entorhinal cortex of TgF344-AD male rats who were exposed to AIE, compared to stationary AIE males (*p* = 0.033; see Figure 6K). There was a trend toward a significant effect of Exercise overall, with voluntary exercise decreasing the number of plaques in the entorhinal cortex of male AD (*p* = 0.057). There was no main effect of AIE on plaque count in the entorhinal cortex of male rats (*p* = 0.71). There was a significant main effect of Exercise on plaque size, with exercise increasing the size of plaques in the entorhinal cortex of male AD rats (*F*[1, 33] = 4.48, *p* = 0.042; see Figure 6L). There was no main effect of AIE on plaque size in the entorhinal cortex of male rats (*p* = 0.70). Exercise resulted in a decrease in percentage of area covered compared to stationary controls (*F*[1, 30] = 5.14, *p* = 0.031; see Figure 6M): Exercise decreased the percentage of area covered in AIE- male rats (*p* = 0.013), an effect not seen water control male AD rats.

### 3.9 Correlations between behavior and histology

**FIGURE 7:**
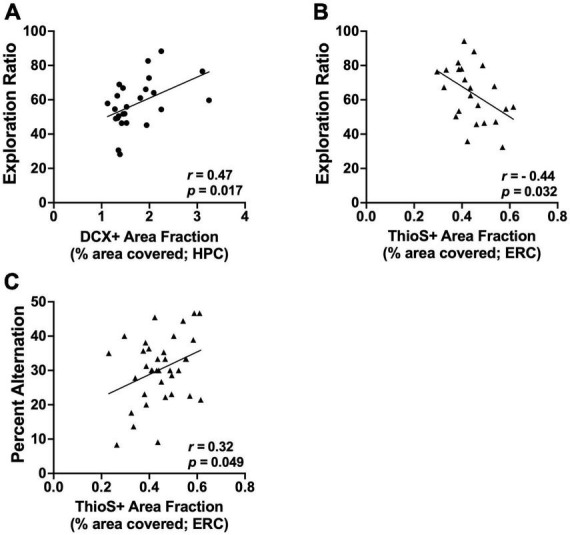
Significant correlations between brain measure and behavior. **(A)** Exploration ratios on NOP positively correlate with DCX+ area fraction in the HPC of female wild-type rats (*p* = 0.017). **(B)** Exploration ratios on NOP negatively correlate with the percentage of the ERC covered by amyloid plaques (ThioS+ staining) in male rats (*p* = 0.032). **(C)** Percent alternation scores on spontaneous alternation positively correlate with the percentage of the ERC covered by amyloid plaques (ThioS+ staining) in male rats (*p* = 0.049). Circle data points represent female rats; triangle data points represent male rats.

Correlations with NOP: There was a positive correlation between exploration ratios on NOP and DCX+ area fraction in the dentate gyrus, but only in wild-type female rats (*r* = 0.47, *p* = 0.01; see Figure 7A). There were no correlations between NOP and DCX expression found in male or AD rats (all *p*’s > 0.14). There were no correlations between performance on NOP and amyloid burden in the hippocampus of either sex.

There was a negative correlation between exploration ratios on NOP and ThioS+ area fraction in the entorhinal cortex of male AD rats (*r* = −0.45, *p* = 0.03; see Figure 7B); as amyloid burden increased in the entorhinal cortex, performance on the NOP task declined. There was no correlation between exploration ratios and ThioS+ area fraction in the entorhinal cortex of female rats.

Correlations between brain changes and spontaneous alternation: There were no correlations between ChAT+ expression in the MS/DB and performance on the spontaneous alternation task in either sex (all *p*’s > 0.42). In addition, there were no correlations between DCX+ count or DCX area fraction and performance on the spontaneous alternation task in either sex (all *p*’s > 0.42). Finally, were no correlations between performance on the spontaneous alternation task and amyloid plaque count, size, or area fraction in the hippocampus of either sex (all *p*’s > 0.15).

In male rats, the percentage of the entorhinal cortex covered by amyloid plaques positively correlated with performance on the spontaneous alternation task (*r* = 0.33, *p* = 0.05; see Figure 7C). Spontaneous alternation scores did not correlate with the number of plaques or the size of plaques in the entorhinal cortex of male rats (*p*’s > 0.08). There were no correlations between performance on the spontaneous alternation task and amyloid plaque count, size, or area fraction in the entorhinal cortex of female rats (all *p*’s > 0.08).

## 4 Conclusion

The main findings of this study are: (1) Males, regardless of genotype or ethanol exposure, run significantly less than female rats. (2) Four weeks of voluntary wheel running recovers spatial working memory deficits assessed by spontaneous alternation selectively in female rats following heavy binge-like ethanol exposure in adolescence. (3) There is sex-specific exercise-induced recovery of unique pathological markers. In AIE-AD female rats, exercise improves the integration of recently born neurons, whereas in AIE-AD males exercise decreases amyloid burden in both the entorhinal cortex and hippocampus. (4) The number of neurons expressing the cholinergic phenotype was not affected by AD transgenes in either sex, but AIE exposure, selectively in female rats, reduced the expression of the cholinergic phenotype. (5) Rather than exercise enhancing the expression of the cholinergic phenotype in neurons, it reduced it in both sexes. Taken together, these findings are unique and shed light on the sexual dimorphism in how developmental ethanol exposure alters AD-related symptomology and neuropathology.

### 4.1 Behavioral consequences of developmental ethanol exposure and AD transgenes and exercise-induced recovery

Exercise has emerged as a profound manipulation to improve cognitive functioning and neuropathological outcomes in several neurodegenerative disorders, including alcohol-related brain pathology (Karoly et al., 2013; Hall and Savage, 2016; Barton et al., 2017; Maynard et al., 2018; Macht et al., 2020) and AD (García-Mesa et al., 2011; Rodríguez et al., 2011; Lin et al., 2015; Morris et al., 2017; Jia et al., 2019). Like other studies, we found a profound sex difference in voluntary wheel running. Other studies have found that this sex difference in running exists even following gonadectomy (Basso and Morrell, 2017), whereas estradiol increases running in both male and female rats (Krentzel et al., 2020). However, despite sex differences in running, both male and female young mice had an increased BDNF response to voluntary wheel running, and decreased EtOH consumption (Gallego et al., 2015). We observed sex-dependent effects of exercise that could be driven by differences in the amount of wheel running. “Dosing” exercise can be complicated in a voluntary exercise paradigm. Despite this, we chose voluntary exercise because voluntary exercise is a more effective intervention than forced treadmill exercise to improve spatial learning ability in aging rats, particularly female aged rats (Belviranli and Okudan, 2022).

The sex-specific acceleration of AIE-induced cognitive deficits in the TgF344-AD model found in in our previous study (Reitz et al., 2024) was replicated in this experiment: Female TgF344-AD rats exposed to AIE had impaired spatial working memory, as indicated by a lower percentage of alternations in the spontaneous alteration task. Voluntary wheel running for 4 weeks at 6-months of age recovered this impairment. Male rats did not differ by exposure or genotype in the stationary conditions, yet following exercise, TgF344-AD AIE exposed rats performed worse than wild-type AIE male rats. The lack of a robust exercise effect in male rats could be due to the reduced amount of exercise in males.

Performance on NOP, a task of pattern separation and spatial memory, was impaired at 6-month of age in all female groups— except for wild-type water controls. Voluntary wheel running recovered this impairment in both TgF344-AD water controls and AIE-exposed female rats, but not wild-type AIE-exposed rats. A similar pattern was observed in male rats, except that TgF344-AD AIE male rats were not impaired on NOP when raised in the stationary condition. It appears that there is potential “high” performing group and a “low” performing group in stationary male TgF344-AD water controls (see Figure 2F), although a higher-powered study would be needed to clarify this phenomenon. A previous study investigating the behavioral phenotype of the TgF344-AD model found that a small percentage (20%) of aged TgF344-AD rats (18–21 months old) perform similarly to young adult wild-type rats (6–8 months old) on a spatial working memory and flexibility task (Bac et al., 2023). On the same task, however, about half of the 6–8 month old TgF344-AD rats were impaired compared to age-matched wild-type rats (Bac et al., 2023). It was suggested that the combination of neuroinflammatory signals and tau kinases drives the emergence of cognitive decline in the TgF344-AD.

It is worth noting that although there were no sex differences found in BEC levels following AIE exposure, TgF344-AD rats did exhibit higher BECs than wild-type Fischer 344 rats when blood was tested one hour following ethanol gavage. This effect of genotype on ethanol metabolism was also found in a previous study using the same AIE protocol (Reitz et al., 2024), although neither this study nor previous studies have investigated ethanol metabolic pathways in the TgF344-AD model. Future studies should analyze metabolic factors such as hepatic alcohol dehydrogenase in this rodent model of AD and consider their potential role in AIE-induced cognitive deficits.

### 4.2 Sex-specific changes in AD-pathological markers as a function of developmental ethanol exposure and exercise

Levels of ChAT+ expressing neurons in the MS/DB, DCX+ expressing neurons in the dentate gyrus of the hippocampus, and ThioS+ amyloid plaques in the hippocampus and entorhinal cortex were assessed to gain insight into the neurobiological foundations of the cognitive changes due to genotype, AIE exposure, and voluntary exercise. Considering the crucial role of the cholinergic system on learning and memory (Maurer and Williams, 2017; Solari and Hangya, 2018), as well as its role in AD (Beach et al., 2000; Ramos-Rodriguez et al., 2013; Hampel et al., 2018), the number of ChAT+ producing neurons in the MS/DB were analyzed. Exposure to AIE decreased ChAT+ expression in the MS/BD of female rats, but not male rats. Surprisingly, voluntary exercise selectively decreased the expression of ChAT+ in AIE exposed wild-type and TgF344-AD rats of both sexes, but the ChAT+ expression in the water controls of both genotypes remained unchanged. Previous research found a decrease in basal forebrain ChAT neuronal expression in young wild type male rodent models of AIE, with exercise during or shortly after AIE recovering the loss (Vetreno et al., 2014, 2018, 2020). The timing of exercise may be critical: The current study placed the exercise program 5-months post AIE treatment. Early work that used NGF infusion as a way to rescue cholinergic neurons found reduced effectiveness as time since pathology passed (Hagg et al., 1988). Thus, the late exposure of exercise in the current study was not as effective as earlier exposure in rescuing the cholinergic profile following AIE.

Using the total number of DCX+ neurons in the dentate gyrus (quantification of cell bodies), as a measure of adult neurogenesis, previous research has found a persistent decrease due to AIE (Vetreno and Crews, 2015; Vetreno et al., 2018; Macht et al., 2020; Reitz et al., 2021). While neurogenesis has been studied in other models, only one study has utilized DCX staining in the TgF344-AD model and they found a significant decrease in GCL DCX when analyzed alongside 5-ethynyl-2′-deoxyuridine (EdU; Morrone et al., 2020). In the current experiment, female TgF344-AD rats did exhibit a decrease in DCX+ neurons compared to wild-type female rats, but there were no additional effects of AIE or exercise. However, when analyzing the percentage of total GCL area covered by DCX+ neurons (includes both cell bodies and dendritic branching), that represents the successful integration of these adult born neurons, there is a significant decrease due to AIE exposure in both female and male rats. Interestingly, this AIE-induced deficit was recovered by exercise in female TgF344-AD rats, but this effect was not observed in wild-type rats. In contrast, in male rats, the AIE-induced deficit was only recovered by exercise in the wild-type rats and not the TgF344-AD rats. These conflicting results could be due, in part, from sex-based differences in the hyperexcitability in the dentate gyrus of TgF344-AD rats (Goodman et al., 2021; Smith et al., 2022). As pattern separation is highly dependent on hippocampal neurogenesis (Sahay et al., 2011; Johnston et al., 2016; Reitz et al., 2021), we ran a correlation on NOP performance and DCX expression in the GCL. Notably, NOP exploration ratios positively correlate with percentage of GCL area covered by DCX+ neurons, suggesting that reduced integration of adult born neurons into hippocampal circuity may contribute to the cognitive decline and recovery in this model following AIE and AD transgenes.

One of the key hallmarks of AD pathology is amyloid plaques, which are typically found starting at 6-months of age in the TgF344-AD model (Cohen et al., 2013). We expected to find an increase in amyloid plaque burden when exposure to ethanol had occurred in adolescence, due to the subsequent disruptions in the cholinergic pathways, signaling cascades, and neurogenesis in the hippocampus that follow AIE (Jahn, 2013; Vetreno et al., 2014, 2018; Vetreno and Crews, 2015; Fernandez and Savage, 2017; Zhu et al., 2017; Hampel et al., 2018; Kinney et al., 2018; Crews et al., 2019; Swartzwelder et al., 2019; Reitz et al., 2021). Using other rodent models of AD, previous research has established that heavy ethanol consumption does result in the increase in amyloid-beta and plaque formation in the cortex and hippocampus (Hoffman et al., 2019; Day et al., 2023). In the current study, there were no changes in ThioS+ plaque size, plaque count, or percent of area covered in the hippocampus or entorhinal cortex of female TgF344-AD rats. This suggests that amyloid plaque burden is not driving the acceleration of cognitive decline in female TgF344-AD rats exposed to AIE. In male rats, however, amyloid burden is uniquely affected by AIE exposure and exercise. Exercise decreased total plaque count and percentage of area covered in the hippocampus or entorhinal cortex of AIE male TgF344-AD rats; exercise did not affect plaque size, count, or area covered in water control TgF344-AD rats. These data suggest that although AIE does not accelerate or increase amyloid beta plaque formation in the TgF344-AD model at 6 months of age, it does contribute to the creation of malleable amyloid beta plaques that can be altered by exercise later in life.

We did find that ThioS+ burden in the entorhinal cortex negatively correlated with NOP and spontaneous alternation performance in male rats. Cortical levels of amyloid beta have been shown to correlate with greater cognitive deficits and heightened risk of progression to AD, but only in patients with MCI and in natural aging (Stevens et al., 2022). Additionally, there is a relatively large proportion of elderly adults (30%) that have comparable levels of cortical amyloid beta seen in patients with AD, yet are cognitively healthy and do not shown signs of cognitive impairment associated with AD (Rowe et al., 2010; Villemagne et al., 2013). It is possible that in prodromal stages of AD, tau accumulation may serve as a mediator between amyloid accumulation and eventual cognitive decline (Hanseeuw et al., 2019). A previous study using the TgF344-AD model has suggested that pTau levels in the hippocampus can predict spatial working memory performance in both male and female rats more successfully and reliably than levels of amyloid beta, and that amyloid beta and neuroinflammation precede tauopathy (Bac et al., 2023). The 7-month endpoint for this experiment is earlier than the expected onset of neurofibrillary tangles in the TgF344-AD model (16 months old; Cohen et al., 2013). The analysis of accumulated tau before tangle formation alongside the analysis of amyloid plaque burden in the entorhinal cortex may provide insight into the relationship between plaque burden in the cortex and associated cognitive decline.

## 5 Summary

Overall, it appears that binge-type alcohol exposure during adolescence alters the trajectory of AD-related impairment on hippocampal dependent spatial tasks, particularly in female rats. Rats with the TgF344-AD genotype performed worse on spontaneous alternation when they had been exposed to AIE, compared to their sex-matched water controls. This deficit in spontaneous alternation was rescued in female rats with four weeks of voluntary wheel running exercise, while exercise exposed a behavioral deficit in male rats. Exposure to AIE also produced deficits in remembering object location, that was not rescued by exercise in wild-type rats, yet exercise was able to recover the deficits in TgF344-AD rats of both sexes. There was also sex-specific effect on markers of brain pathology: In female rats exposed to alcohol that also had AD transgenes, exercise recovered the reductions in the area covered by doublecortin staining within the subgranular zone of the hippocampus. This supports that concept that exercise improves the integration of newly born neurons in severe disease states and improves spatial memory. In contrast, in male rats with AD transgenes exposed to alcohol exercise reduced plaque number and area covered by plaques in both the hippocampus and entorhinal cortex. However, this reduction in plaques did not translate into behavioral recovery. It is possible that at the early-mid disease state captured in this model, we are at the nexus of compensation and damage.

## Data availability statement

The raw data supporting the conclusions of this article will be made available by the authors, without undue reservation.

## Ethics statement

The animal study was approved by the IACUC Binghamton University State University of New York. The study was conducted in accordance with the local legislation and institutional requirements.

## Author contributions

NR: Conceptualization, Data curation, Formal analysis, Investigation, Methodology, Validation, Visualization, Writing – original draft, Writing – review & editing. PN: Formal analysis, Visualization, Writing – review & editing. LS: Conceptualization, Funding acquisition, Supervision, Validation, Visualization, Writing – original draft, Writing – review & editing.
